# Trends of Large for Gestational Age and Macrosomia and Their Mediating Effect on the Association Between Diabetes Mellitus and Obstetric Hemorrhage

**DOI:** 10.1111/mcn.70000

**Published:** 2025-02-16

**Authors:** Peiran Chen, Yi Mu, Yanxia Xie, Yanping Wang, Zheng Liu, Mingrong Li, Juan Liang, Jun Zhu

**Affiliations:** ^1^ National Office for Maternal and Child Health Surveillance of China, West China Second University Hospital Sichuan University Chengdu Sichuan China; ^2^ Department of Obstetrics, West China Second University Hospital Sichuan University Chengdu Sichuan China; ^3^ Key Laboratory of Birth Defects and Related Diseases of Women and Children (Sichuan University), Ministry of Education Chengdu Sichuan China

**Keywords:** diabetes mellitus in pregnancy, large for gestational age, macrosomia, mediating effect, obstetric haemorrhage, trend

## Abstract

This study aimed to determine the prevalence of large for gestational age (LGA) and macrosomia in China from 2012 to 2021 and explore if LGA and macrosomia mediate the relationship between diabetes mellitus in pregnancy (DIP) and obstetric haemorrhage. The overall annual change rate (ACR) was calculated, and stratification analysis was performed. Mediation analysis assessed the influence of LGA and macrosomia in the association between DIP and obstetric haemorrhage. The nationwide prevalence of LGA and macrosomia was 15.8% and 6.8%, respectively. The ACR for LGA was 0.71% (95% CI, 0.66%–0.76%); pre‐2016, −0.44% (95% CI, −0.63% to −0.25%); post‐2016, −0.29% (95% CI, −0.39% to −0.19%). The ACR for macrosomia was −1.17% (95% CI, −1.24% to −1.09%); pre‐2016, −0.73% (95% CI, −1.03% to −0.43%); post‐2016, −2.42% (95% CI, −2.59% to −2.26%). In western and rural area, the ACR for LGA was 1.94% (95% CI, 1.84%–2.05%) and 1.81% (95% CI, 1.73%–1.89%), and LGA was increasing among these women in the post‐2016 period. About 7.0% of pregnant women had DIP, and the LGA and macrosomia prevalences among women with DIP were 23.7% and 10.0%, respectively. In the mediation analysis, the total excess risk associated with DIP on obstetric haemorrhage was approximately 0.21 and the proportion mediated by LGA and macrosomia was 12.10% and 11.81%, respectively. In rural areas, the proportion mediated by LGA and macrosomia was amplified to 18.34% and 16.40%. Macrosomia rates declined steadily, but LGA rates increased slightly in disadvantaged areas. LGA and macrosomia mediated the association between DIP and obstetric haemorrhage, and the mediating effect intensified in rural regions. Addressing LGA warrants management for at‐risk fetuses.

## Summary

- The nationwide prevalence of macrosomia in China has declined in recent years.
- Controlling fetal weight at the threshold of LGA remained a challenge, particularly for women from disadvantaged regions.
- Macrosomia or LGA may mediate the association between diabetes mellitus and obstetric haemorrhage.

## Graphical abstract

LGA and macrosomia are in different decreasing rates in China. Both LGA and macrosomia can work as the mediator in the association between diabetes mellitus in pregnancy and obstetric haemorrhage, and the mediating effects are similar.

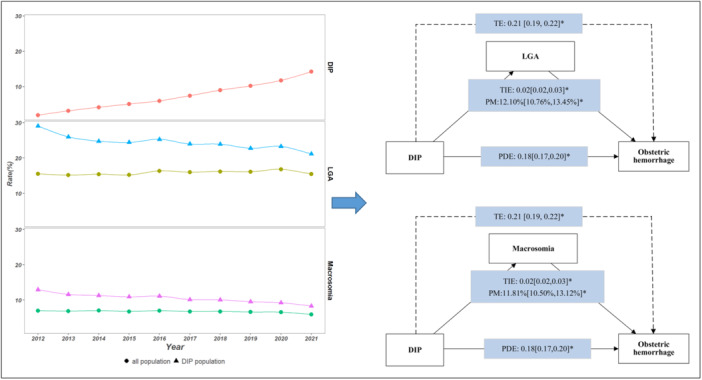

## 1 Introduction

Fetus/fetal weight is an indicator of fetus/fetal health and growth in utero. Maldevelopment of intrauterine fetus/fetal growth may result in fetus/fetal growth restriction (FGR) and excessive weight gain. The measurements of fetus/fetal overweight include large for gestational age (LGA) and macrosomia. One population‐based study showed that the prevalence of macrosomia and LGA in China between 2013 and 2017 was 4.21% and 8.65%, respectively (Zeng et al. 2023). The reported macrosomia rate in China is lower than that in the United States (8.07%, 2017), close to that in Brazil (5.4%, 2014) and higher than that in Korea (2.5%, 2020) (Hur 2023; Nascimento et al. 2017; Salihu et al. 2020). The implementation of China's ‘two‐child’ policy in 2016 potentially altered the proportion of multiparous women, possibly impacting the prevalence of fetus/fetal overweight. However, the nationwide trend in fetus/fetal overgrowth after 2017 remains relatively unknown. Some uncertainty also remains regarding specific trends across different regions and socioeconomic statuses in China.

Abnormal birth weights are also associated with multiple factors, such as gestational diabetes mellitus, gestational weight gain and maternal body mass index, parity and parental stature (Chen et al. 2023; He et al. 2023; Lei et al. 2020; G. Li et al. 2014; Mazzone et al. 2023; Yuan et al. 2022). A recently released national standard limited the gestational weight gain for pregnant women who were overweight before pregnancy and diagnosed with diabetes mellitus (National Health Commission of the People's Republic of China 2023). Among all these fetus/fetal overweight risk factors, diabetes mellitus can also be a risk factor for adverse maternal outcomes (Ende et al. 2021; Tavera et al. 2022; Weissmann‐Brenner et al. 2012). On the other hand, abnormal birth weight may adversely affect maternal health, such as an increased risk of postpartum haemorrhage (PPH) (Beta et al. 2019; Liu et al. 2021; Qian et al. 2023; Saevarsdottir et al. 2023). However, understanding the intricate relationship between diabetes mellitus in pregnancy (DIP) and adverse maternal outcomes is complex. While DIP may directly impact adverse maternal outcomes or through fetus/fetal overweight indirectly, few studies have substantiated this association. Assessing the mediating effect of fetus/fetal overweight in the causal pathway of DIP and adverse outcomes, and quantifying its impact, could offer new insights for developing interventions to mitigate the health risks associated with DIP by managing fetus/fetal weight.

This study utilized data collected from a comprehensive national hospital‐based surveillance system with the primary objective of illustrating the prevalence and temporal trends of LGA and macrosomia across the nation and within different regions from 2012 to 2021. Additionally, a mediation analysis was employed to quantitatively assess the impact of DIP on the occurrence of obstetric haemorrhage through fetus/fetal overweight.

## 2 Methods

### 2.1 Study Participants

This observational study evaluated pregnant women who delivered singleton live births at 438 hospitals registered in the National Maternal Near‐Miss Surveillance System (NMNMSS) between January 1, 2012, and December 31, 2021. The methodology for hospital selection and data quality assurance procedures were detailed in a previous report (Deng et al. 2021). The NMNMSS collected obstetric information such as birth date, delivery method, fetus/fetal status, birth weight and sex of the baby whenever a birth occurred. Other maternal sociodemographic and demographic data, such as gestational age, marital status, educational status, parity, prenatal examinations, and maternal complications, were recorded for each patient admitted to the obstetric department of each of the sampled hospitals.

For the descriptive analysis of fetus/fetal overweight prevalence and trends, the study considered women who delivered singleton live births at gestational ages ranging from 28 to 42 weeks. Exclusions comprised cases involving multiple births or those lacking information on birth weight or the baby's sex. Since fetus/fetal overweight may be affected by several factors, and DIP is one of the main factors, the prevalence of DIP and the prevalence of fetus/fetal overweight among women with DIP were also estimated.

In the mediation analysis, the sample excluded cases involving prenatal maternal complications or medical conditions other than DIP and singleton live births where the infant's weight fell below the 10th percentile of the birth weight standard (small for gestational age, SGA) from the descriptive sample.

### 2.2 Variable Definitions

The study categorized fetus/fetal overweight as encompassing both LGA and macrosomia. LGA was defined as a singleton live birth where the infant's birth weight exceeded the 90th percentile of the national birth weight standard corresponding to the baby's sex and gestational age (National Health Commission of the People's Republic of China 2022). Additionally, to ensure comparability on an international scale, LGA was also defined based on the INTERGROWTH‐21st standard in a sensitivity analysis (Villar et al. 2014). A kappa value was calculated to assess consistency between the two standards. Macrosomia was classified as a singleton live birth where the infant's birth weight surpassed 4000 g. In this study, the term ‘DIP’ encompassed both pre‐existing diabetes mellitus and diabetes mellitus diagnosed during pregnancy.

For this study, obstetric haemorrhages encompassed conditions such as placental abruption, uterine atony, uterine rupture and PPH.

### 2.3 Statistical Analysis

The prevalences of macrosomia and LGA were calculated nationwide and in different subgroups, such as by region (eastern/central/western), area (north/south), location (urban/rural) and maternal sociodemographic characteristics over the study period. Given the NMNMSS's focus on larger urban hospitals, the macrosomia and LGA prevalences were adjusted based on the population's sampling distribution from China's 2010 Census, as previously outlined (Zhu et al. 2016). The annual change rate (ACR) was derived from a Poisson regression model with a robust variance estimator, using time in years as the explanatory, continuous variable. China's ‘two‐child’ policy implementation in 2016 may have impacted the burden of macrosomia and LGA. Stratification (pre‐2016 and post‐2016) analysis of the ACR estimates was also performed.

Multivariable logistic regressions were built to explore the possible factors impacting macrosomia and LGA. Except for DIP, other variables including birth year, region, area, location, hospital level, maternal age, maternal education level, marital status, gestational age, prenatal visits, scarred uterus, fetus/fetal sex and other complications were taken as covariates simultaneously.

Mediation analysis, widely employed for exploring causality between multiple exposures and outcomes, was used to examine the mediating role of LGA and macrosomia in the link between DIP and obstetric haemorrhage in this study (Amadou et al. 2022; Johnson et al. 2021; Jung 2021; Thorpe et al. 2022). This modelling approach decomposites the overall effect of exposure on the excess relative risk of an outcome into four components: the effect of exposure without the mediator (CDE), the interactive effect when the mediator was kept at its absence of exposure level (INT‐ref), the mediated interaction (INT‐med) and the pure indirect effect (PIE) (Discacciati et al. 2018; VanderWeele 2014, 2016).

In this study, the total effect of the excess risk of obstetric haemorrhage associated with DIP was categorized as the pure direct effect (PDE, which means the excess risk of obstetric haemorrhage associated with DIP directly) and the total indirect effect (TIE, which means the excess risk of obstetric haemorrhage associated with DIP but working through LGA or macrosomia indirectly). The PDE was estimated as the sum of the CDE and the INT‐ref. The TIE was estimated as the sum of the PIE and the INT‐med. The proportion mediated (PM) was calculated as the proportion of the total indirect effect that took the amount of the total effect. We estimated DIP and the mediator's (LGA and macrosomia) effect on obstetric haemorrhage using logistic regression (Model 1). We also estimated the DIP's effect on LGA and macrosomia using logistic regression (Model 2).

(1) $$\text{log }\text{it}\left\{P\left(Y=1|\alpha ,m,c\right)\right\}=\theta_{0}+\theta_{1}\alpha +\theta_{2}m+\theta_{3}\alpha m+\theta_{4}^{\prime }c\;\left(\text{Model }1\right),$$

(2) $$\text{log }\text{it}\left\{P\left(M=1|\alpha ,c\right)\right\}=\beta_{0}+\beta_{1}\alpha +\beta_{2}^{\prime }c\;\left(\text{Model }2\right).$$

Model 1 analysed the association between DIP ($\theta_{1}\alpha$) and obstetric haemorrhage, considering the mediate effect of LGA or macrosomia ($\theta_{2}m$) and its interaction with DIP ($\theta_{3}\alpha m$), given covariates on the condition of *C* = *c* ($\theta_{4}^{\prime }c$). Model 2 analysed the association between DIP and LGA or macrosomia ($\beta_{1}\alpha$) given covariates on the condition of *C* = *c* ($\beta_{2}^{\prime }c$). Covariates adjusted for in the model were time in years, gestational age and maternal demographic factors (region, area, location, hospital level, maternal age, maternal education level, marital status, parity, and prenatal visits).

A significance level of two‐sided *p* < 0.05 was considered statistically significant. All analyses were conducted using SAS statistical software version 9.4 (SAS Institute Inc., Cary, NC, USA), Stata version 16.0 (Stata Corp., TX, USA) and R 3.6.0 (R Foundation for Statistical Computing, Vienna, Austria).

### 2.4 Ethics

This study was approved by the ethics committee of the Hospital (protocol ID, 2012008).

## 3 Results

A total of 13,009,361 women were evaluated in our analysis of the prevalence and trends of LGA and macrosomia. The demographic characteristics of the participants are shown in Table 1. The crude nationwide prevalences were 16.0% for LGA and 6.6% for macrosomia. After adjusting for sampling distribution, the LGA and macrosomia prevalences were 15.8% and 6.8%, respectively (Table 2). The sampling distribution adjusted rates of LGA and macrosomia varied between different subgroups. The LGA rate was 17.1% in the east, 17.0% in the central region, 12.5% in the west, 19.6% in the north, 13.4% in the south, 15.1% in women aged < 35 years, 20.9% in women with advanced maternal age, 13.5% in primiparas and 18.4% in multiparas (Supporting Information S1: Table 1). The macrosomia rates were 7.1% in the east, 7.6% in the central region, 5.1% in the west, 9.3% in the north, 5.1% in the south, 6.6% in women aged < 35 years, 8.0% in women with advanced maternal age, 6.1% in primiparas and 7.4% in multiparas (Supporting Information S1: Table 2).

**Table 1:** Demographic characters of enrolled women by fetus/fetal birth year (*n* = 13,009,361).

		Years			
Characters	Overall	2012	2015	2018	2021
Region					
East	3749,686 (28.82%)	373,989 (29.67%)	362,639 (28.96%)	373,396 (28.39%)	287,920 (28.11%)
Central	5,207,066 (40.03%)	500,631 (39.71%)	492,100 (39.29%)	534,460 (40.63%)	406,796 (39.71%)
West	4,052,609 (31.15%)	385,955 (30.62%)	397,607 (31.75%)	407,531 (30.98%)	329,574 (32.18%)
Area					
North	5,184,506 (39.85%)	508,470 (40.34%)	458,113 (36.58%)	518,793 (39.44%)	396,321 (38.69%)
South	7,824,855 (60.15%)	752,105 (59.66%)	794,233 (63.42%)	796,594 (60.56%)	627,969 (61.31%)
Location					
Urban	7,941,389 (61.04%)	686,568 (54.46%)	714,457 (57.05%)	839,189 (63.80%)	694,332 (67.79%)
Rural	5,067,972 (38.96%)	574,007 (45.54%)	537,889 (42.95%)	476,198 (36.20%)	329,958 (32.21%)
Hospital level					
Level 1 or Level 2	7,176,044 (55.16%)	771,250 (61.18%)	738,773 (58.99%)	685,601 (52.12%)	493,125 (48.14%)
Level3	5,833,317 (44.84%)	489,325 (38.82%)	513,573 (41.01%)	629,786 (47.88%)	531,165 (51.86%)
Maternal age					
< 35 years	11,277,449 (86.69%)	1,141,989 (90.59%)	1,108,382 (88.50%)	1,109,077 (84.32%)	850,843 (83.07%)
Above 35 years	1,731,912 (13.31%)	118,586 (9.41%)	143,964 (11.50%)	206,310 (15.68%)	173,447 (16.93%)
Maternal education					
Below college	7,541,398 (57.97%)	871,352 (69.12%)	816,979 (65.24%)	699,975 (53.21%)	469,716 (45.86%)
College or above	5,210,908 (40.06%)	363,848 (28.86%)	406,698 (32.47%)	592,932 (45.08%)	536,550 (52.38%)
Unknown	257,055 (1.98%)	25,375 (2.01%)	28,669 (2.29%)	22,480 (1.71%)	18,024 (1.76%)
Prenatal visit					
≥ 8 times	7,335,877 (56.39%)	536,021 (42.52%)	628,727 (50.20%)	840,807 (63.92%)	712,522 (69.56%)
< 8 times	5,232,616 (40.22%)	696,729 (55.27%)	591,524 (47.23%)	431,564 (32.81%)	255,377 (24.93%)
Unknown	440,868 (3.39%)	27,825 (2.21%)	32,095 (2.56%)	43,016 (3.27%)	56,391 (5.51%)
Parity					
Primipara	7,054,783 (54.23%)	820,549 (65.09%)	694,326 (55.44%)	633,922 (48.19%)	524,198 (51.18%)
Multipara	5,951,030 (45.74%)	438,578 (34.79%)	557,831 (44.54%)	681,260 (51.79%)	499,944 (48.81%)
Unknown	3548 (0.03%)	1448 (0.11%)	189 (0.02%)	205 (0.02%)	148 (0.01%)
Marital status					
Single/divorce	182,669 (1.40%)	17,616 (1.40%)	18,875 (1.51%)	17,097 (1.30%)	18,851 (1.84%)
Married	12,824,456 (98.58%)	1,242,639 (98.58%)	1,233,240 (98.47%)	1,298,062 (98.68%)	1,005,363 (98.15%)
Unknown	2236 (0.02%)	320 (0.03%)	231 (0.02%)	228 (0.02%)	76 (0.01%)
Scared uterus					
No	10,799,531 (83.01%)	1,124,918 (89.24%)	1,053,938 (84.16%)	1,044,170 (79.38%)	830,895 (81.12%)
Yes	2,186,190 (16.80%)	124,578 (9.88%)	198,023 (15.81%)	270,649 (20.58%)	193,158 (18.86%)
Unknown	23,640 (0.18%)	11,079 (0.88%)	385 (0.03%)	568 (0.04%)	237 (0.02%)

**Table 2:** Stratification (pre‐2016 and post‐2016) of annual change rate (ACR, %) with its 95% confidence interval of LGA and macrosomia among singleton births.^a^

		ACR of LGA rate (%)^b^				ACR of macrosomia rate (%)^b^		
Characters	LGA rate (%)	Overall	Pre‐2016	Post‐2016	Macrosomia rate (%)	Overall	Pre‐2016	Post‐2016
Overall	15.8 (15.8, 15.8)	0.71 (0.66, 0.76)*	−0.44 (−0.63, −0.25)*	−0.29 (−0.39, −0.19)*	6.8 (6.7, 6.8)	−1.17 (−1.24, −1.09)*	−0.73 (−1.03, −0.43)*	−2.42 (−2.59, −2.26)*
90th–97th	9.9 (9.9, 9.9)	0.87 (0.80, 0.93)*	−0.20 (−0.46, 0.05)	−0.02 (−0.16, 0.11)	—	—	—	—
> 97th	5.9 (5.9, 6.0)	0.44 (0.36, 0.53)*	−0.82 (−1.15, −0.49)*	−0.72 (−0.90, −0.55)*	—	—	—	—
4000–4500 g	—	—	—	—	6.0 (6.0, 6.0)	−1.08 (−1.16, −1.00)*	−0.75 (−1.07, −0.43)*	−2.22 (−2.40, −2.05)*
4500–5000 g	—	—	—	—	0.7 (0.7, 0.7)	−1.70 (−1.95, −1.45)*	−0.28 (−1.25, 0.70)	−3.59 (−4.11, −3.06)*
> 5000 g	—	—	—	—	0.1 (0.1, 0.1)	−2.46 (−3.10, −1.82)*	−2.83 (−5.33, −0.27)*	−6.34 (−7.68, −4.97)*
Region	—	—						
East	17.1 (17.1, 17.2)	−0.08 (−0.16, 0.00)	−1.53 (−1.86, −1.21)*	−1.18 (−1.36, −1.01)*	7.1 (7.1, 7.1)	−1.85 (−1.98, −1.71)*	−2.42 (−2.94, −1.91)*	−2.96 (−3.25, −2.67)*
Central	17.0 (17.0, 17.1)	0.65 (0.58, 0.72)*	−0.27 (−0.56, 0.02)	−0.21 (−0.36, −0.06)*	7.6 (7.6, 7.7)	−1.23 (−1.35, −1.12)*	−0.68 (−1.13, −0.24)*	−2.58 (−2.82, −2.34)*
West	12.5 (12.5, 12.6)	1.94 (1.84, 2.05)*	1.25 (0.83, 1.67)*	1.10 (0.89, 1.31)*	5.1 (5.1, 5.1)	−0.09 (−0.26, 0.08)	2.34 (1.66, 3.02)*	−1.09 (−1.44, −0.73)*
Area								
North	19.6 (19.5, 19.6)	0.67 (0.60, 0.74)*	0.66 (0.39, 0.94)*	−0.45 (−0.59, −0.31)*	9.2 (9.2, 9.3)	−1.35 (−1.45, −1.24)*	0.62 (0.20, 1.03)*	−2.98 (−3.20, −2.76)*
South	13.4 (13.3, 13.4)	0.87 (0.80, 0.94)*	−0.62 (−0.89, −0.35)*	0.06 (−0.08, 0.21)	5.1 (5.1, 5.1)	−0.73 (−0.85, −0.62)*	−0.82 (−1.27, −0.38)*	−1.44 (−1.69, −1.20)*
Location								
Urban	16.9 (16.9, 16.9)	−0.88 (−0.94, −0.83)*	−1.84 (−2.08, −1.61)*	−2.10 (−2.22, −1.98)*	6.5 (6.4, 6.5)	−2.12 (−2.21, −2.02)*	−1.84 (−2.24, −1.45)*	−3.83 (−4.03, −3.63)*
Rural	15.0 (14.9, 15.0)	1.81 (1.73, 1.89)*	0.37 (0.08, 0.66)*	1.30 (1.13, 1.46)*	7.0 (7.0, 7.0)	−0.24 (−0.35, −0.12)*	0.02 (−0.40, 0.45)	−0.96 (−1.21, −0.70)*
Hospital level								
Level 1 or Level 2	15.3 (15.3, 15.4)	1.41 (1.35, 1.48)*	0.13 (−0.13, 0.38)	0.71 (0.57, 0.85)*	7.0 (7.0, 7.0)	−0.48 (−0.58, −0.38)*	−0.27 (−0.64, 0.11)	−1.39 (−1.61, −1.17)*
Level 3	16.8 (16.7, 16.8)	−0.85 (−0.92, −0.79)*	−1.88 (−2.17, −1.60)*	−2.07 (−2.21, −1.93)*	6.3 (6.3, 6.3)	−2.15 (−2.27, −2.04)*	−1.73 (−2.21, −1.25)*	−3.86 (−4.09, −3.62)*
Maternal age								
< 35 years	15.1 (15.1, 15.1)	0.41 (0.36, 0.46)*	−0.96 (−1.16, −0.75)*	−0.42 (−0.53, −0.31)*	6.6 (6.6, 6.6)	−1.24 (−1.32, −1.15)*	−1.02 (−1.34, −0.70)*	−2.45 (−2.63, −2.27)*
≥ 35 years	20.9 (20.9, 21.0)	0.23 (0.12, 0.35)*	1.69 (1.18, 2.21)*	−0.67 (−0.89, −0.45)*	8.0 (8.0, 8.0)	−2.10 (−2.30, −1.90)*	0.40 (−0.46, 1.26)	−2.88 (−3.27, −2.49)*
Maternal education								
Below college	15.3 (15.3, 15.3)	1.44 (1.37, 1.50)*	1.69 (1.18, 2.21)*	0.86 (0.72, 1.00)*	6.8 (6.8, 6.8)	−0.39 (−0.49,‐ 0.29)*	−0.21 (−0.58, 0.16)	−1.29 (−1.52, −1.07)*
College or above	16.9 (16.8, 16.9)	−1.27 (−1.34, −1.20)*	−2.67 (−2.98, −2.35)*	−2.10 (−2.25, −1.96)*	6.7 (6.7, 6.8)	−2.78 (−2.90, −2.66)*	−2.54 (−3.06, −2.02)*	−3.91 (−4.15, −3.67)*
Marital status								
Single/divorce	10.6 (10.4, 10.7)	2.05 (1.55, 2.55)*	−2.67 (−2.98, −2.35)*	3.22 (2.17, 4.28)*	3.9 (3.8, 4.0)	1.63 (0.78, 2.48)*	−2.49 (−5.80, 0.94)	1.32 (−0.44, 3.11)
Married	15.9 (15.9, 15.9)	0.70 (0.65, 0.74)*	−0.39 (−0.58, −0.20)*	−0.29 (−0.39, −0.19)*	6.8 (6.8, 6.8)	−1.19 (−1.27, −1.11)*	−0.70 (−1.00, −0.39)*	−2.42 (−2.59, −2.26)*
Parity								
Primipara	13.5 (13.4, 13.5)	−0.82 (−0.89, −0.75)*	−2.54 (−2.80, −2.28)*	−1.18 (−1.33, −1.02)*	6.1 (6.1, 6.1)	−1.47 (−1.57, −1.36)*	−1.14 (−1.55, −0.74)*	−3.01 (−3.25, −2.77)*
Multipara	18.4 (18.4, 18.4)	0.89 (0.83, 0.96)*	0.09 (−0.19, 0.37)	0.19 (0.06, 0.32)*	7.4 (7.4, 7.5)	−1.66 (−1.77, −1.55)*	−1.72 (−2.16, −1.27)*	−2.04 (−2.26, −1.82)*
Prenatal visit								
≥ 8 times	16.3 (16.2, 16.3)	0.02 (−0.04, 0.09)	−1.98 (−2.25, −1.71)*	−0.80 (−0.93, −0.68)*	6.8 (6.8, 6.9)	−1.51 (−1.61, −1.41)*	−2.29 (−2.72, −1.86)*	−2.64 (−2.85, −2.44)*
< 8 times	15.3 (15.3, 15.4)	1.19 (1.11, 1.27)*	0.41 (0.14, 0.69)*	0.36 (0.19, 0.54)*	6.7 (6.7, 6.7)	−0.82 (−0.95, −0.70)*	0.36 (−0.07, 0.78)	−2.11 (−2.39, −1.82)*
Gestational age								
28–31 weeks	28.9 (28.5, 29.2)	−5.48 (−5.86, −5.10)*	−5.67 (−7.05, −4.27)*	−5.81 (−6.66, −4.94)*	—	—	—	—
32–36 weeks	21.9 (21.8, 22.0)	−1.67 (−1.84, −1.51)*	−2.47 (−3.12, −1.82)*	−2.05 (−2.41, −1.69)*	—	—	—	—
≥ 37 weeks	15.4 (15.4, 15.5)	0.92 (0.87, 0.97)*	−0.22 (−0.43, −0.02)*	−0.15 (−0.25, −0.04)*	—	—	—	—

^a^ Coefficient estimates were adjusted for the sampling distribution of the population.

^b^ Crude ACR.

* *p* < 0.01 with robust estimator.

**Figure 1:**
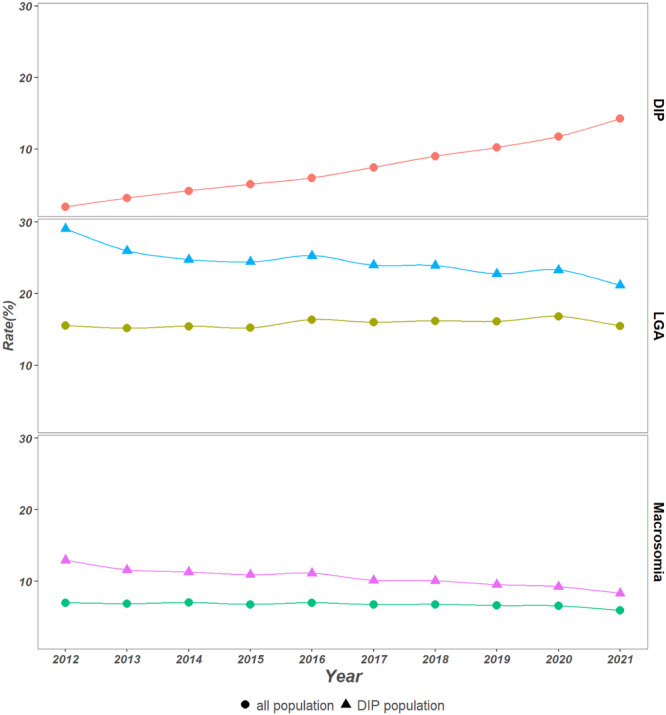
Prevalence of DIP, LGA and macrosomia between 2012 and 2021.

The prevalence rate of LGA was 15.5% in 2012 and 15.5% in 2021, and the ACR was 0.71% (95% confidence interval [CI], 0.66%–0.76%). In the stratification analysis, the pre‐2016 ACR was −0.44% (95% CI, −0.63% to −0.25%) and the post‐2016 ACR was −0.29% (95% CI, −0.39% to −0.19%). Regarding different regions and areas, the ACRs were −0.08% (95% CI, −0.16% to 0.00%) in the eastern region, 0.65% (95% CI, 0.58%–0.72%) in the central region, and 1.94% (95% CI, 1.84%–2.05%) in the western region. The ACR was −0.88% (95% CI, −0.94% to −0.83%) for the urban zone and 1.81% (95% CI, 1.73%–1.89%) for the rural zone. For different levels of education, the ACR was 1.44% (95% CI, 1.37%–1.50%) for maternal educational levels below college, and −1.27% (95% CI, −1.34% to −1.20%) for mothers with college‐level educations or higher. The ACR was 0.02% (95% CI, −0.04% to 0.09%) for those who had adequate prenatal visits (≥ 8 times) and 1.19% (95% CI, 1.11%–1.27%) for those with inadequate prenatal visits (< 8 time). Women from subgroups of west, rural, and non‐tertiary hospitals, or multiparas were in an increasing trend of LGA in the post‐2016 period (Table 2). The prevalence rate of macrosomia was 7.0% in 2012 and 5.9% in 2021, and the ACR was −1.17% (95% CI, −1.24% to −1.09%). In the stratification analysis, the pre‐2016 ACR was −0.73% (95% CI, −1.03% to −0.43%) and the post‐2016 ACR was −2.42% (95% CI, −2.59% to −2.26%). In different birth weight subgroups, higher birth weights decreased faster from 2012 to 2021. And the prevalence of macrosomia showed a decreasing trend in the overall and stratification analyses in different subgroups (Table 2). The trend of LGA and macrosomia were showed in Figure 1. The LGA rate and macrosomia rate in each year from 2012 to 2021 are shown in Supporting Information S1: Tables 1 and 2.

In the multivariable regression analysis, women from rural area, with advanced maternal age, with higher education levels, multiparas and those with scarred uteri or DIP had an increased risk of fetus/fetal macrosomia or LGA. Among these risk factors, DIP was one of the most relevant factors for LGA (adjusted odds ratio [aOR] was 1.56 [95% CI, 1.56–1.57]) and macrosomia (aOR 1.89 [95% CI, 1.87–1.90]) (Supporting Information S1: Table 3). From 2012 to 2021, about 7.0% of pregnant women were diagnosed with DIP and the ACR was 20.87% (95% CI, 20.79%–20.96%). Among DIP women, the LGA rate was 23.7%, which was decreased from 29.0% in 2012 to 21.1% in 2021, and the ACR was −2.36% [95% CI, −2.48% to −2.20%]. The prevalence rate of macrosomia was 10.0%, which was decreased from 12.9% in 2012 to 8.3% in 2021 and the ACR was −4.03% [95% CI, −4.26% to −3.81%].

**Figure 2:**
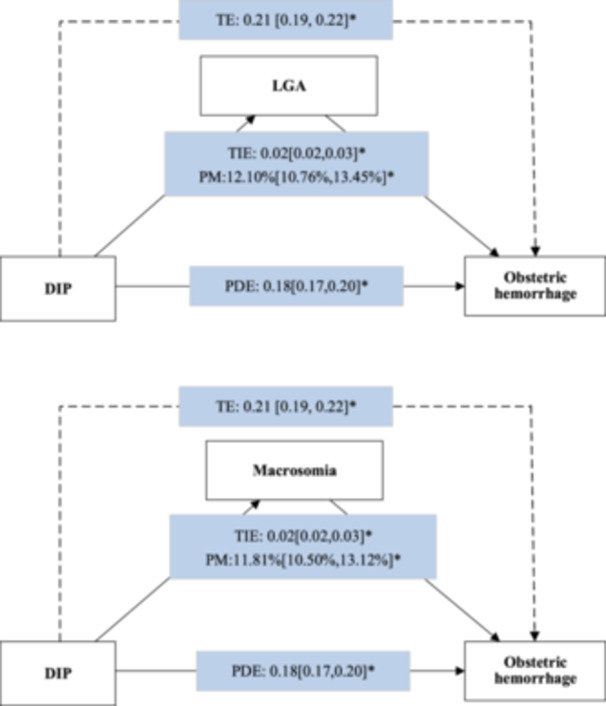
LGA and macrosomia as the mediator in the association between DIP and obstetric haemorrhage. DIP, diabetes mellitus in pregnancy; eRR, excess relative risk; LGA, large for gestational age; PDE, pure direct effect; PM, proportion mediated; TE, total effect, TE = PDE + TIE; TIE, total indirect effect. *p < 0.01.

After excluding women who gave birth to SGA infants, a total of 10,712,534 women were enrolled in our mediation analysis, and 796,409 were diagnosed with DIP (Supporting Information S1: Table 4). In the overall analysis, when LGA was taken as the mediator, the adjusted total effect of DIP in terms of raising the excess risk of obstetric haemorrhage was 0.21 (95% CI, 0.19–0.22), and the proportion of the total effect that mediated by LGA was 12.10% (95% CI, 10.76%–13.45%). When macrosomia was taken as the mediator, the adjusted total effect of DIP in terms of increased risk of obstetric haemorrhage was 0.21 (95% CI, 0.19–0.22), and the proportion of the total effect that mediated by macrosomia was 11.81% (95% CI, 10.50%–13.12%) (Figure 2). From 2012 to 2019, the proportion mediated by LGA and macrosomia could account for roughly 8%–15% of the total effect of DIP in terms of raising the excess risk of obstetric haemorrhage. From 2020, while the total effect and direct effect of DIP continued to decrease, the indirect effect of DIP remained stable, resulting in the proportion mediated by LGA and macrosomia increasing to 30% (Supporting Information S1: Figure 1). The unadjusted results are shown in Supporting Information S1: Table 5.

In our subgroup analysis, the excess risk of DIP on obstetric haemorrhage that mediated by LGA was greater in the northern (PM, 15.25%; 95% CI, 12.03%–18.48%) and rural regions (PM, 18.34%; 95% CI, 15.05%–21.63%). In addition, the excess risk of DIP on obstetric haemorrhage that mediated by macrosomia was also greater in the northern (PM, 16.49%; 95% CI, 13.25%–19.74%) and rural regions (PM, 16.40%; 95% CI, 13.28%–19.53%; Table 3).

**Table 3:** LGA and macrosomia as the mediator through the association between DIP and obstetric haemorrhage in different subgroups.

		LGA		Macrosomia	
Subgroups	Effect decomposition	eRR	PM	eRR	PM
Region					
East					
	TE	0.21 [0.18, 0.23]		0.21 [0.18, 0.23]	
	PDE	0.18 [0.16, 0.21]		0.19 [0.16, 0.21]	
	TIE	0.02 [0.02, 0.03]	11.36% [9.24%, 13.47%]*	0.02 [0.02, 0.03]	10.76% [8.75%, 12.77%]*
Central					
	TE	0.17 [0.14, 0.19]		0.17 [0.15, 0.20]	
	PDE	0.15 [0.12, 0.17]		0.14 [0.12, 0.17]	
	TIE	0.02 [0.02, 0.03]	13.79% [10.68%, 16.90%]*	0.03 [0.02, 0.03]	15.19% [12.03%, 18.35%]*
West					
	TE	0.22 [0.19, 0.25]		0.22 [0.20, 0.25]	
	PDE	0.20 [0.17, 0.22]		0.20 [0.17, 0.23]	
	TIE	0.03 [0.02, 0.03]	11.66% [9.51%, 13.80%]*	0.02 [0.02, 0.03]	10.36% [8.31%, 12.42%]*
Area					
North					
	TE	0.18 [0.15, 0.20]		0.18 [0.15, 0.21]	
	PDE	0.15 [0.13, 0.18]		0.15 [0.13, 0.18]	
	TIE	0.03 [0.02, 0.03]	15.25% [12.03%, 18.48%]*	0.03 [0.02, 0.03]	16.49% [13.25%, 19.74%]*
South					
	TE	0.20 [0.19, 0.22]		0.21 [0.19, 0.22]	
	PDE	0.18 [0.17, 0.20]		0.19 [0.17, 0.20]	
	TIE	0.02 [0.02, 0.02]	10.35% [8.90%, 11.79%]*	0.02 [0.02, 0.02]	9.34% [7.99%, 10.68%]*
Location					
Urban	TE	0.18 [0.16, 0.20]		0.18 [0.16, 0.20]	
	PDE	0.16 [0.14, 0.18]		0.16 [0.15, 0.18]	
	TIE	0.02 [0.02, 0.02]	10.87% [9.26%, 12.49%]*	0.02 [0.02, 0.02]	10.67% [9.14%, 12.21%]*
Rural					
	TE	0.24 [0.21, 0.27]		0.24 [0.21, 0.27]	
	PDE	0.20 [0.16, 0.23]		0.20 [0.17, 0.23]	
	TIE	0.04 [0.04, 0.05]	18.34% [15.05%, 21.63%]*	0.04 [0.03, 0.05]	16.40% [13.28%, 19.53%]*
Maternal education					
Below college					
	TE	0.24 [0.21, 0.26]		0.24 [0.22, 0.26]	
	PDE	0.20 [0.18, 0.23]		0.21 [0.19, 0.23]	
	TIE	0.03 [0.03, 0.04]	13.85% [11.81%, 15.88%]*	0.03 [0.03, 0.03]	12.61% [10.68%, 14.54%]*
College or above					
	TE	0.18 [0.16, 0.19]		0.18 [0.16, 0.20]	
	PDE	0.16 [0.14, 0.18]		0.16 [0.14, 0.18]	
	TIE	0.02 [0.02, 0.02]	10.56% [8.72%, 12.40%]*	0.02 [0.02, 0.02]	10.85% [9.02%, 12.68%]*
Maternal marital status					
Single/divorce					
	TE	0.06 [−0.07, 0.19]		0.05 [−0.08, 0.18]	
	PDE	0.04 [−0.09, 0.17]		0.03 [−0.10, 0.16]	
	TIE	0.02 [−0.00, 0.04]	30.87% [−42.51%, 104.2%]	0.02 [0.01, 0.04]	46.23% [−69.93%, 162.4%]
Married					
	TE	0.21 [0.19, 0.22]		0.21 [0.19, 0.22]	
	PDE	0.18 [0.17, 0.20]		0.18 [0.17, 0.20]	
	TIE	0.03 [0.02, 0.03]	12.01% [10.67%, 13.35%]*	0.02 [0.02, 0.03]	11.69% [10.38%, 13.00%]*
Maternal age					
< 35 years					
	TE	0.22 [0.20, 0.23]		0.22 [0.20, 0.23]	
	PDE	0.19 [0.17, 0.20]		0.19 [0.17, 0.21]	
	TIE	0.03 [0.03, 0.03]	13.64% [12.03%, 15.25%]*	0.03 [0.03, 0.03]	13.23% [11.66%, 14.79%]*
≥ 35 years					
	TE	0.18 [0.15, 0.21]		0.18 [0.15, 0.21]	
	PDE	0.17 [0.13, 0.20]		0.17 [0.14, 0.20]	
	TIE	0.02 [0.01, 0.02]	8.43% [6.00%, 10.85%]*	0.01 [0.01, 0.02]	8.25% [5.82%, 10.69%]*
Parity					
Primipara					
	TE	0.24 [0.21, 0.26]		0.24 [0.22, 0.26]	
	PDE	0.21 [0.19, 0.23]		0.21 [0.19, 0.24]	
	TIE	0.03 [0.02, 0.03]	11.41% [9.76%, 13.05%]*	0.02 [0.02, 0.03]	9.52% [8.02%, 11.02%]*
Multipara					
	TE	0.17 [0.15, 0.19]		0.17 [0.15, 0.19]	
	PDE	0.15 [0.12, 0.17]		0.14 [0.12, 0.16]	
	TIE	0.02 [0.02, 0.03]	13.44% [11.05%, 15.84%]*	0.03 [0.02, 0.03]	15.69% [13.15%, 18.23%]*

Abbreviations: DIP, diabetes mellitus in pregnancy; eRR, excess relative risk; LGA, large for gestational age; PDE, pure direct effect; PM, proportion mediated; TE, total effect, TE = PDE + TIE; TIE, total indirect effect.

* *p* < 0.01.

In our sensitivity analysis, the mediation analysis of the definition of LGA according to the ‘INTERGROWTH‐21st’ standard was close to that of LGA defined using the national standard (Supporting Information S1: Table 6). The LGA results for the two definitions were consistent, with both sharing a kappa value of 0.9444 (95% CI, 0.9442–0.9447, *p* = 0.0003).

## 4 Discussion

To our knowledge, this study is the first to illustrate the nationwide prevalence and trends of LGA and macrosomia in China after the implementation of the ‘universal two‐child’ policy. It also aimed to quantitatively assess LGA or macrosomia as the mediator in the excess risk of DIP on obstetric haemorrhage. Between 2012 and 2021, the nationwide prevalences of macrosomia and LGA were 6.8% and 15.8%, respectively. While macrosomia exhibited a decreasing trend over the study duration, LGA rates increased slightly in disadvantage areas. Our findings suggest that LGA or macrosomia could mediate the excess risk of obstetric haemorrhage associated with DIP. Furthermore, the rising trend of LGA and the mediating effect elucidated by LGA and macrosomia emphasize the need for increased attention to birth weight control, particularly for DIP women in rural regions.

The nationwide LGA and macrosomia rates in this study were within the range of what has been reported in previous studies (Harvey, van Elburg, and van der Beek 2021; Zeng et al. 2023). However, these rates have been derived mainly from partial studies that did not have nationwide representativeness. One study using data from the National Free Pre‐Pregnancy Check‐up Project (NFPCP) between 2013 and 2017 showed that the nationwide LGA and macrosomia prevalences were 8.65% and 4.21%, respectively (Zeng et al. 2023)—both of which were much lower than the rates we found in our study. Possible reasons for these differences are as follows. First, the inclusion and exclusion criteria for participants varied between the two studies. In our study, we restricted the participants to women who had singleton live births at gestational ages between 28 and 42 weeks. This is in accordance with the World Health Organization's (WHO's) definition of live births (Lawn et al. 2011). In the other study, singleton births between 22 and 28 weeks gestational age and stillbirths were also included. Such births are generally less likely to be overweight. Second, the compositions of the study years and participants differed. In our study, we included women who had singleton live births between 2012 and 2021, rather than the period between 2013 and 2017 used in the other study. With the establishment of China's ‘universal two‐child’ policy in 2016, a higher proportion of women with advanced maternal ages and with multiparity was observed (H. T. Li et al. 2019; Liang et al. 2018; Zhang et al. 2021). A longer study period after the fertility policy change was included in our study, which may have captured more women of advanced maternal ages and multiparas. Prior studies have shown that advanced maternal age and multiparity were risk factors for fetus/fetal overgrowth (Teguete, Maiga, and Leppert 2012; Walsh and McAuliffe 2012). The larger amount of multiparas and women of advanced maternal age may have contributed to a higher proportion of LGA and macrosomia observed in this study.

In addition, there were still differences between the trend of macrosomia and LGA. We found a decreasing trend for macrosomia in the overall and stratification analyses. In some subgroups, the macrosomia even decreased faster in the post‐2016 period than in the pre‐2016 period. The decreasing trend for macrosomia from 2012 to 2021 is consistent with the findings of Zeng et al. (Zeng et al. 2023). Meanwhile, although LGA was on a decreasing trend in the pre‐2016 and post‐2016 periods, LGA still showed an overall increasing trend from 2012 to 2021. This discrepancy may be related to the instant increase of LGA in 2016 and a constantly high level of LGA from 2016 to 2021. Additionally, unlike macrosomia, an accelerated decreasing trend of LGA was not observed in the post‐2016 period. In subgroups such as women from western, rural and non‐tertiary hospitals or multiparas, an increasing trend of LGA was even observed in the overall or post‐2016 period. The difference between the trends in macrosomia and LGA may be attributable to several reasons. First, macrosomia can be considered as an extreme form of LGA, and it may be easier to control extreme overgrowth. This hypothesis was confirmed by the results of this study where birth weights above the 97th percentile of the standard or above 5000 g decreased the most between 2012 and 2021. Second, LGA standards are complex, unlike macrosomia, which has a universal standard. Healthcare providers can easily diagnose macrosomia and provide treatment once the fetus/fetal weight reaches approximately 4000 g. The standard for LGA is based on sex and gestational age, and the threshold for LGA differs accordingly. Evaluating LGA according to this standard requires advanced ultrasonic instruments, professional knowledge, experience, and techniques in maternal healthcare—making it difficult to put this standard into practical use. Coupled with increased multiparas associated with the implementation of the ‘universal two‐child’ policy, a lack of timely and effective fetus/fetal weight control ultimately led to an upward trend in the prevalence of LGA in disadvantaged areas (Ren et al. 2022). However, researchers are now calling for the adoption of LGA standards, owing to their sensitivity (Harvey, van Elburg, and van der Beek 2021). Our results suggest a challenge in fetus/fetal weight control at the threshold of LGA, particularly for women from disadvantaged regions.

Previous studies have reported an increased risk of macrosomia and LGA fetuses among women with DIP, where maternal hyperglycaemia increased fetus/fetal utilization of glucose and adipose tissue and eventually led to fetus/fetal overweight (Kc, Shakya, and Zhang 2015; Sgayer et al. 2024). This was in agreement with our study findings that among the covariates we considered in the multivariable analysis of the risk factors for LGA and macrosomia, DIP was one of the most correlated factors. Moreover, the rising trend of DIP in China has aroused wide concern among the public. With the efforts to decrease the adverse effects of DIP (including guidelines for the diagnosis and treatment of hyperglycaemia during pregnancy, guidelines for pregnancy weight gain, maternal nutritional clinics, and pregnancy risk assessment and management) (Department of Obstetrics and Gynecology of Chinese Medical Association 2018; Department of Obstetrics and Gynecology of Chinese Medical Association, & Gestational diabetes cooperative group of Chinese Perinatal Medicine Association 2014; National Health and Family Planning Commission 2016; National Health and Family Planning Commission General Office 2017), the prevalence rates of LGA and macrosomia were in a downward trend in the context of DIP increasing. However, the constantly heavier burden of LGA and macrosomia among women with DIP still cannot neglected. In our study, the prevalence rates of LGA and macrosomia among DIP women were 21.1% and 8.3% in 2021, respectively, which were almost 1.4 times that among the overall population. Our results suggest that LGA and macrosomia were byproducts of DIP and may have an adverse effect on maternal health as well.

The atypically large fetuses are usually associated with an increased risk of baby stuck in the birth canal, birth asphyxia, shoulder dystocia, prolonged labour, perineal tears, uterine atony, uterine rupture, heavy bleeding, and PPH (Beta et al. 2019; Kc, Shakya, and Zhang 2015; Said and Manji 2016). If severe obstetric bleeding is not treated timeously, it may increase the need of blood transfusion; some may even progress to a serious pregnancy outcomes, such as hysterectomies, ICU admissions, near‐misses, and maternal deaths (Rocha Filho et al. 2015). Our overall and mediation result in each year from 2012 to 2021 showed that LGA and macrosomia act as mediators of the association between DIP and obstetric haemorrhage, and fetus/fetal overweight is an important factor in increasing the excess risk of obstetric haemorrhage in DIP. The mediating analysis showed that when the total effect and direct effect of DIP do not decline any further, the proportion mediated by LGA and macrosomia will increase. This suggests that the diagnosis and treatment of DIP cannot ignore the importance of controlling fetus/fetal birthweight.

Besides, the mediating analysis in different subgroups showed that in rural regions where certain unappropriate nutritional behaviours were common (Ma et al. 2020), the excess risk of DIP through LGA and macrosomia could escalate further. Interestingly, the subpopulation in which a larger proportion of the effect mediated by fetus/fetal overgrowth was generally consistent with the subgroups of women where an increasing trend of LGA was observed. This overlap calls for more attention to be paid to fetus/fetal weight control in pregnant women from rural areas during prenatal visits.

This study had some key limitations worth noting. First, both the LGA and macrosomia rates were calculated from hospital‐based surveillance results, and the LGA and macrosomia rates may be associated with the bias from the selection of the surveillance hospital. However, the population sample distribution from China's 2010 Census was adopted to adjust for the estimated LGA and macrosomia rates to decrease this bias. Second, information concerning pre‐pregnancy weights or weight gains during pregnancy was not collected in this study. Although failure to adjust for the effect of the maternal body mass index and weight gain in pregnancy in the model may have caused some bias in the estimation of DIP's effect, the importance of the effect of DIP on macrosomia and LGA has been confirmed in previous studies and clinical practice. Further studies analysing the mediating effect of fetus/fetal overgrowth could consider adjusting for the effect of maternal weight. Besides, to minimize the effect of confounders, we adjusted the possible factors related to LGA and macrosomia, such as maternal age, prenatal visits, maternal education levels, parity, and scarred uteri in this study. In addition, we excluded women with prenatal maternal complications or medical conditions other than DIP in the mediation analysis to avoid potential confounding brought by other maternal complications.

## 5 Conclusion

While the nationwide occurrence of macrosomia has shown a steady decline, the attention given to LGA has been comparatively less. With the implementation of the ‘universal two‐child’ policy, the LGA risk may potentially increase. LGA or macrosomia appears to mediate the heightened risk of obstetric haemorrhage associated with DIP. More significantly, this mediating effect may be greatly amplified in rural regions. The rising prevalence of LGA infants among women from disadvantaged areas introduces a new challenge in managing birth weights within the LGA range. Given the similar mediating effect of LGA and macrosomia and more sensitive LGA diagnosing standards, it is essential not to disregard fetus/fetal overweight that surpasses LGA. Early detection and close intervention for fetus/fetal weight management prove particularly advantageous whenever a risk of LGA emerges, especially for women residing in rural areas.

## Author Contributions

All authors have contributed to the conducting of this study. P.C., Y.M., J.L. and J.Z. designed the study with contributions from all authors. P.C. and Y.M. did the statistical analysis with support from J.L., Y.X. and J.Z. P.C. and Y.M. prepared the first draft and all authors contributed to critical interpretation of the results and development of the report.

## Conflicts of Interest

The authors declare no conflicts of interest.

## Supporting information

Supporting information.

## Data Availability

The data that support the findings of this study are available from National Office for Maternal and Child Health Surveillance of China but restrictions apply to the availability of these data, which were used under license for the current study, and so are not publicly available. Data are however available from the authors upon reasonable request and with permission of the National Health Commission of the People's Republic of China.
