# Supplementary material for: Trends of Large for Gestational Age and Macrosomia and Their Mediating Effect on the Association Between Diabetes Mellitus and Obstetric Hemorrhage
Source: Matern Child Nutr. 2025 Feb 16;21(3):e70000. doi: 10.1111/mcn.70000 (PMC12150131; doi:10.1111/mcn.70000)
Supplement: Supplementary file 1 — Supporting information. [file MCN-21-e70000-s001.docx]

**Supplementary**

The incidence of LGA is 15.4%, using the “INTERGROWTH-21^st^” standard. The kappa value for using the “INTERGROWTH-21^st^” standard and using the national fetal growth standard is 0.9444 (95%CI is 0.9442, 0.9447), P=0.0003.

**Supplementary Table 1. Prevalence rate (%) with its 95% confidence interval of LGA among singleton live births from 2012 to 2021 in China***

| **Characters** | **Year** | | | | | | | | | |
| --- | --- | --- | --- | --- | --- | --- | --- | --- | --- | --- |
|  | **2012** | **2013** | **2014** | **2015** | **2016** | **2017** | **2018** | **2019** | **2020** | **2021** |
| **Overall** | 15.5(15.5,15.6) | 15.1(15.1,15.2) | 15.4(15.4,15.5) | 15.2(15.1,15.3) | 16.4(16.3,16.4) | 16.0(15.9,16.1) | 16.2(16.1,16.3) | 16.1(16.0,16.2) | 16.8(16.7,16.9) | 15.5(15.4,15.6) |
| 90^th^~97^th^ | 9.6(9.6,9.7) | 9.4(9.4,9.5) | 9.6(9.6,9.7) | 9.5(9.4,9.6) | 10.2(10.1,10.2) | 10.0(9.9,10.0) | 10.1(10.1,10.2) | 10.1(10.1,10.2) | 10.5(10.5,10.6) | 9.8(9.7,9.9) |
| >97^th^ | 5.9(5.9,6.0) | 5.7(5.7,5.8) | 5.8(5.8,5.9) | 5.7(5.7,5.8) | 6.2(6.1,6.2) | 6.0(6.0,6.0) | 6.1(6.0,6.1) | 6.0(6.0,6.0) | 6.3(6.2,6.3) | 5.7(5.6,5.7) |
| **Region** |  |  |  |  |  |  |  |  |  |  |
| east | 17.5(17.4,17.6) | 16.8(16.7,17.0) | 16.8(16.6,16.9) | 16.6(16.5,16.8) | 18.0(17.9,18.1) | 17.1(16.9,17.2) | 17.2(17.1,17.3) | 17.1(17.0,17.2) | 17.8(17.6,17.9) | 16.1(16.0,16.2) |
| central | 16.7(16.6,16.8) | 16.3(16.2,16.4) | 16.7(16.6,16.8) | 16.5(16.3,16.6) | 17.6(17.5,17.7) | 17.1(17.0,17.2) | 17.2(17.1,17.3) | 17.3(17.2,17.4) | 18.0(17.9,18.2) | 16.8(16.6,16.9) |
| west | 11.6(11.4,11.7) | 11.6(11.5,11.7) | 12.0(11.9,12.1) | 11.9(11.8,12.0) | 12.6(12.5,12.7) | 12.8(12.7,13.0) | 13.3(13.2,13.5) | 13.2(13.1,13.3) | 13.9(13.7,14.0) | 12.9(12.8,13.0) |
| **Area** |  |  |  |  |  |  |  |  |  |  |
| north | 19.0(18.9,19.1) | 18.7(18.6,18.8) | 19.1(19.0,19.2) | 19.3(19.2,19.4) | 20.2(20.1,20.3) | 19.9(19.8,20.0) | 20.0(19.9,20.1) | 19.9(19.8,20.0) | 20.6(20.4,20.7) | 19.1(19.0,19.2) |
| south | 13.2(13.1,13.2) | 12.7(12.7,12.8) | 12.8(12.8,12.9) | 12.9(12.8,12.9) | 13.8(13.7,13.8) | 13.5(13.4,13.5) | 13.8(13.7,13.8) | 13.6(13.5,13.6) | 14.4(14.3,14.5) | 13.2(13.1,13.3) |
| **Location** |  |  |  |  |  |  |  |  |  |  |
| urban | 17.8(17.6,17.9) | 17.1(17.0,17.2) | 16.9(16.8,17.0) | 16.8(16.7,16.9) | 17.6(17.5,17.7) | 17.1(17.0,17.2) | 17.1(17.0,17.2) | 16.5(16.4,16.6) | 16.8(16.7,16.9) | 15.3(15.2,15.4) |
| rural | 14.2(14.1,14.2) | 13.9(13.8,14.0) | 14.4(14.3,14.5) | 14.2(14.1,14.2) | 15.4(15.3,15.5) | 15.1(15.0,15.2) | 15.4(15.3,15.5) | 15.7(15.6,15.8) | 16.8(16.7,16.9) | 15.7(15.6,15.8) |
| **Hospital level** |  |  |  |  |  |  |  |  |  |  |
| level 1 or level 2 | 14.7(14.6,14.8) | 14.4(14.3,14.5) | 14.8(14.8,14.9) | 14.6(14.5,14.7) | 15.8(15.7,15.9) | 15.5(15.4,15.6) | 15.7(15.6,15.8) | 15.9(15.8,16.0) | 16.9(16.8,17.0) | 15.7(15.6,15.8) |
| level 3 | 17.6(17.5,17.7) | 17.0(16.8,17.1) | 16.8(16.6,16.9) | 16.6(16.5,16.7) | 17.4(17.3,17.5) | 17.0(16.9,17.1) | 17.0(16.9,17.2) | 16.5(16.4,16.6) | 16.6(16.5,16.7) | 15.2(15.1,15.3) |
| **Maternal age** |  |  |  |  |  |  |  |  |  |  |
| <35 yrs | 15.1(15.0,15.1) | 14.6(14.6,14.7) | 14.9(14.8,14.9) | 14.5(14.4,14.6) | 15.5(15.5,15.6) | 15.2(15.2,15.3) | 15.3(15.2,15.3) | 15.3(15.2,15.3) | 15.9(15.9,16.0) | 14.6(14.5,14.7) |
| ≥35 yrs | 20.2(20.0,20.5) | 20.0(19.8,20.3) | 21.1(20.8,21.3) | 21.0(20.8,21.3) | 22.3(22.1,22.5) | 20.0(19.8,20.1) | 21.6(21.4,21.8) | 21.2(21.0,21.4) | 21.7(21.5,21.9) | 20.1(19.9,20.3) |
| **Maternal education** |  |  |  |  |  |  |  |  |  |  |
| below college | 14.7(14.6,14.7) | 14.4(14.3,14.5) | 14.8(14.8,14.9) | 14.5(14.5,14.6) | 15.7(15.6,15.8) | 15.5(15.4,15.6) | 15.7(15.6,15.8) | 15.8(15.7,15.9) | 16.9(16.8,17.0) | 15.8(15.7,15.9) |
| college or above | 18.3(18.1,18.4) | 17.4(17.3,17.6) | 17.0(16.9,17.1) | 16.9(16.7,17.0) | 17.5(17.4,17.6) | 16.9(16.8,17.0) | 16.9(16.8,17.0) | 16.5(16.4,16.6) | 16.7(16.6,16.8) | 15.2(15.1,15.3) |
| **Marital status** |  |  |  |  |  |  |  |  |  |  |
| single/divorce | 10.7(10.3,11.2) | 9.6(9.2,10.0) | 10.1(9.7,10.5) | 9.3(8.9,9.7) | 10.2(9.8,10.6) | 10.6(10.2,11.1) | 10.6(10.2,11.1) | 11.3(10.8,11.8) | 11.8(11.3,12.3) | 11.8(11.4,12.3) |
| married | 15.6(15.5,15.7) | 15.2(15.2,15.3) | 15.5(15.5,15.6) | 15.3(15.2,15.4) | 16.4(16.4,16.5) | 16.1(16.0,16.1) | 16.3(16.2,16.3) | 16.2(16.1,16.2) | 16.9(16.8,17.0) | 15.6(15.5,15.6) |
| **Parity** |  |  |  |  |  |  |  |  |  |  |
| primipara | 14.2(14.1,14.3) | 13.7(13.6,13.7) | 13.6(13.6,13.7) | 13.0(12.9,13.1) | 13.8(13.7,13.8) | 13.1(13.0,13.2) | 13.4(13.3,13.5) | 13.3(13.2,13.4) | 13.5(13.4,13.6) | 12.4(12.3,12.5) |
| multipara | 17.8(17.7,17.9) | 17.4(17.3,17.5) | 17.9(17.8,18.0) | 17.7(17.6,17.8) | 19.0(18.9,19.1) | 18.4(18.3,18.5) | 18.6(18.5,18.7) | 18.6(18.5,18.7) | 19.6(19.5,19.7) | 18.4(18.3,18.5) |
| **Prenatal visit** |  |  |  |  |  |  |  |  |  |  |
| ≥ 8 times | 16.6(16.5,16.7) | 16.0(15.9,16.1) | 15.9(15.8,16.0) | 15.6(15.5,15.7) | 16.7(16.6,16.7) | 16.4(16.3,16.5) | 16.5(16.4,16.5) | 16.3(16.2,16.4) | 16.9(16.8,17.0) | 15.4(15.3,15.5) |
| < 8 times | 14.8(14.7,14.9) | 14.6(14.5,14.6) | 15.0(15.0,15.1) | 14.9(14.8,14.9) | 16.0(15.9,16.1) | 15.5(15.4,15.5) | 15.7(15.6,15.8) | 15.7(15.6,15.8) | 16.7(16.6,16.8) | 15.7(15.6,15.8) |
| **Gestational age** |  |  |  |  |  |  |  |  |  |  |
| 28-31 weeks | 36.7(35.5,37.8) | 34.1(32.9,35.2) | 33.3(32.2,34.4) | 30.4(29.3,31.5) | 29.6(28.6,30.6) | 28.3(27.3,29.3) | 25.1(24.1,26.1) | 24.4(23.4,25.4) | 24.7(23.6,25.7) | 21.2(20.2,22.3) |
| 32-36 weeks | 23.9(23.5,24.2) | 22.8(22.5,23.1) | 22.6(22.2,22.9) | 22.0(21.7,22.4) | 22.5(22.2,22.8) | 21.5(21.1,21.8) | 21.1(20.8,21.5) | 20.9(20.6,21.2) | 21.5(21.2,21.9) | 19.4(19.0,19.7) |
| ≥37 weeks | 15.0(14.9,15.1) | 14.7(14.6,14.7) | 15.0(14.9,15.0) | 14.8(14.7,14.8) | 16.0(15.9,16.0) | 15.6(15.6,15.7) | 15.9(15.8,15.9) | 15.8(15.7,15.9) | 16.5(16.4,16.6) | 15.2(15.1,15.3) |

Excluding stillbirth and birthweight unknown sample, n=13009361.

* rates were adjusted for the sampling distribution of the population;

**Supplementary Table 2. Prevalence rate (%) with its 95% confidence interval of macrosomia among singleton live births from 2012 to 2021 in China***

| **Characters** | **Year** | | | | | | | | | |
| --- | --- | --- | --- | --- | --- | --- | --- | --- | --- | --- |
|  | **2012** | **2013** | **2014** | **2015** | **2016** | **2017** | **2018** | **2019** | **2020** | **2021** |
| **Overall** | 7.0(6.9,7.0) | 6.9(6.8,6.9) | 7.0(7.0,7.1) | 6.8(6.7,6.8) | 7.0(6.9,7.0) | 6.7(6.7,6.8) | 6.8(6.7,6.8) | 6.6(6.6,6.7) | 6.6(6.5,6.6) | 5.9(5.9,6.0) |
| 4000-4500g | 6.2(6.1,6.2) | 6.1(6.0,6.1) | 6.2(6.1,6.2) | 6.0(5.9,6.0) | 6.2(6.1,6.2) | 6.0(5.9,6.0) | 5.9(5.9,6.0) | 5.9(5.8,5.9) | 5.8(5.8,5.9) | 5.3(5.2,5.3) |
| 4500-5000g | 0.7(0.7,0.7) | 0.7(0.7,0.7) | 0.7(0.7,0.7) | 0.7(0.7,0.7) | 0.7(0.7,0.7) | 0.7(0.7,0.7) | 0.7(0.7,0.7) | 0.7(0.6,0.7) | 0.6(0.6,0.7) | 0.6(0.6,0.6) |
| >5000g | 0.1(0.1,0.1) | 0.1(0.1,0.1) | 0.1(0.1,0.1) | 0.1(0.1,0.1) | 0.1(0.1,0.1) | 0.1(0.1,0.1) | 0.1(0.1,0.1) | 0.1(0.1,0.1) | 0.1(0.1,0.1) | 0.1(0.1,0.1) |
| **Region** |  |  |  |  |  |  |  |  |  |  |
| east | 7.7(7.6,7.8) | 7.3(7.3,7.4) | 7.4(7.3,7.4) | 7.1(7.0,7.2) | 7.4(7.3,7.5) | 6.9(6.8,7.0) | 6.9(6.9,7.0) | 6.9(6.8,7.0) | 6.8(6.7,6.9) | 6.0(5.9,6.1) |
| central | 7.9(7.8,8.0) | 7.8(7.7,7.9) | 7.9(7.8,8.0) | 7.7(7.6,7.8) | 7.9(7.9,8.0) | 7.6(7.6,7.7) | 7.6(7.5,7.7) | 7.4(7.4,7.5) | 7.3(7.3,7.4) | 6.7(6.6,6.8) |
| west | 4.9(4.8,5.0) | 5.0(4.9,5.1) | 5.3(5.3,5.4) | 5.2(5.1,5.2) | 5.1(5.1,5.2) | 5.1(5.1,5.2) | 5.3(5.2,5.4) | 5.1(5.0,5.1) | 5.1(5.0,5.2) | 4.7(4.6,4.8) |
| **Area** |  |  |  |  |  |  |  |  |  |  |
| north | 9.5(9.4,9.6) | 9.4(9.3,9.5) | 9.6(9.5,9.7) | 9.6(9.5,9.7) | 9.6(9.5,9.7) | 9.3(9.2,9.4) | 9.3(9.2,9.3) | 8.9(8.9,9.0) | 8.8(8.7,8.9) | 8.0(7.9,8.0) |
| south | 5.3(5.2,5.3) | 5.2(5.1,5.2) | 5.2(5.1,5.2) | 5.1(5.1,5.2) | 5.2(5.2,5.3) | 5.1(5.0,5.1) | 5.2(5.1,5.2) | 5.0(5.0,5.1) | 5.1(5.1,5.2) | 4.7(4.6,4.7) |
| **Location** |  |  |  |  |  |  |  |  |  |  |
| urban | 7.0(6.9,7.1) | 6.7(6.7,6.8) | 6.8(6.7,6.8) | 6.6(6.5,6.6) | 6.8(6.7,6.9) | 6.4(6.4,6.5) | 6.6(6.5,6.6) | 6.2(6.2,6.3) | 6.0(5.9,6.1) | 5.4(5.3,5.4) |
| rural | 7.0(6.9,7.0) | 6.9(6.9,7.0) | 7.2(7.1,7.2) | 6.9(6.8,7.0) | 7.1(7.1,7.2) | 7.0(6.9,7.1) | 7.0(6.9,7.0) | 7.0(6.9,7.1) | 7.1(7.0,7.2) | 6.5(6.5,6.6) |
| **Hospital level** |  |  |  |  |  |  |  |  |  |  |
| level 1 or level 2 | 7.1(7.0,7.1) | 7.0(6.9,7.0) | 7.2(7.1,7.2) | 6.9(6.9,7.0) | 7.2(7.1,7.2) | 7.0(6.9,7.0) | 7.0(6.9,7.0) | 7.0(6.9,7.0) | 7.0(7.0,7.1) | 6.4(6.4,6.5) |
| level 3 | 6.9(6.8,6.9) | 6.5(6.5,6.6) | 6.6(6.6,6.7) | 6.4(6.4,6.5) | 6.6(6.5,6.7) | 6.3(6.2,6.4) | 6.4(6.4,6.5) | 6.1(6.0,6.1) | 5.8(5.7,5.9) | 5.2(5.2,5.3) |
| **Maternal age** |  |  |  |  |  |  |  |  |  |  |
| <35 yrs | 6.8(6.8,6.9) | 6.7(6.7,6.8) | 6.8(6.8,6.9) | 6.6(6.5,6.6) | 6.8(6.7,6.8) | 6.6(6.5,6.6) | 6.6(6.5,6.6) | 6.4(6.4,6.5) | 6.4(6.3,6.4) | 5.7(5.7,5.8) |
| ≥35 yrs | 8.6(8.4,8.8) | 8.3(8.2,8.5) | 8.7(8.5,8.8) | 8.6(8.4,8.8) | 8.6(8.5,8.7) | 7.6(7.5,7.7) | 8.0(7.8,8.1) | 7.7(7.6,7.8) | 7.6(7.5,7.7) | 7.0(6.9,7.1) |
| **Maternal education** |  |  |  |  |  |  |  |  |  |  |
| below college | 6.8(6.8,6.9) | 6.8(6.7,6.8) | 6.9(6.9,7.0) | 6.7(6.6,6.8) | 6.9(6.9,7.0) | 6.8(6.8,6.9) | 6.8(6.7,6.9) | 6.7(6.7,6.8) | 6.8(6.7,6.9) | 6.3(6.2,6.3) |
| college or above | 7.7(7.6,7.8) | 7.3(7.2,7.4) | 7.3(7.2,7.3) | 7.0(6.9,7.1) | 7.1(7.0,7.2) | 6.7(6.6,6.7) | 6.8(6.7,6.8) | 6.5(6.4,6.5) | 6.2(6.2,6.3) | 5.6(5.5,5.6) |
| **Marital status** |  |  |  |  |  |  |  |  |  |  |
| single/divorce | 3.9(3.6,4.1) | 3.6(3.4,3.9) | 3.7(3.5,4.0) | 3.5(3.2,3.8) | 3.8(3.5,4.0) | 4.2(3.9,4.5) | 4.0(3.7,4.3) | 4.2(3.9,4.5) | 4.2(3.9,4.6) | 4.0(3.7,4.3) |
| married | 7.0(7.0,7.1) | 6.9(6.9,7.0) | 7.1(7.0,7.1) | 6.8(6.8,6.9) | 7.0(7.0,7.1) | 6.8(6.7,6.8) | 6.8(6.8,6.9) | 6.6(6.6,6.7) | 6.6(6.5,6.6) | 6.0(5.9,6.0) |
| **Parity** |  |  |  |  |  |  |  |  |  |  |
| primipara | 6.4(6.4,6.5) | 6.2(6.2,6.3) | 6.4(6.4,6.5) | 6.1(6.0,6.2) | 6.3(6.3,6.4) | 6.1(6.0,6.1) | 6.2(6.1,6.2) | 6.0(6.0,6.1) | 5.8(5.7,5.9) | 5.2(5.1,5.2) |
| multipara | 8.0(7.9,8.1) | 7.8(7.7,7.9) | 7.8(7.7,7.9) | 7.5(7.5,7.6) | 7.6(7.6,7.7) | 7.3(7.2,7.4) | 7.3(7.2,7.3) | 7.1(7.1,7.2) | 7.2(7.1,7.3) | 6.7(6.6,6.7) |
| **Prenatal visit** |  |  |  |  |  |  |  |  |  |  |
| ≥ 8 times | 7.4(7.3,7.4) | 7.1(7.0,7.1) | 7.1(7.0,7.1) | 6.8(6.7,6.9) | 7.1(7.0,7.2) | 6.9(6.8,6.9) | 6.9(6.8,6.9) | 6.7(6.7,6.8) | 6.7(6.6,6.7) | 6.0(5.9,6.0) |
| < 8 times | 6.8(6.7,6.8) | 6.7(6.7,6.8) | 7.0(6.9,7.0) | 6.8(6.7,6.8) | 6.9(6.9,7.0) | 6.7(6.6,6.7) | 6.7(6.6,6.7) | 6.5(6.4,6.6) | 6.5(6.4,6.5) | 6.0(6.0,6.1) |

Excluding stillbirth and birthweight unknown, n=13009361.

* rates were adjusted for the sampling distribution of the population;

**Supplementary Table 3. Multifactorial analysis of LGA and Macrosomia**

| **Factors** | | **LGA** | **Macrosomia** |
| --- | --- | --- | --- |
| Year | 2012 | ref | ref |
|  | 2013 | 0.96[0.95,0.97] * | 0.95[0.94,0.96] * |
|  | 2014 | 0.94[0.94,0.95] * | 0.95[0.94,0.96] * |
|  | 2015 | 0.92[0.92,0.93] * | 0.93[0.92,0.94] * |
|  | 2016 | 0.94[0.93,0.95] * | 0.95[0.94,0.96] * |
|  | 2017 | 0.87[0.87,0.88] * | 0.87[0.86,0.88] * |
|  | 2018 | 0.88[0.87,0.88] * | 0.88[0.87,0.89] * |
|  | 2019 | 0.86[0.85,0.86] * | 0.85[0.84,0.86] * |
|  | 2020 | 0.89[0.88,0.89] * | 0.88[0.87,0.89] * |
|  | 2021 | 0.79[0.79,0.80] * | 0.77[0.76,0.78] * |
| Region | east | ref | ref |
|  | central | 0.95[0.95,0.96] * | 0.94[0.94,0.95] * |
|  | west | 0.77[0.77,0.77] * | 0.70[0.69,0.70] * |
| Area | north | ref | ref |
|  | south | 0.63[0.63,0.63] * | 0.55[0.55,0.55] * |
| Location | urban | ref | ref |
|  | rural | 1.03[1.03,1.04] * | 1.08[1.07,1.08] * |
| Hospital level | level 1 or level 2 | 0.98[0.97,0.98] * | 1.00[0.99,1.00] |
|  | level 3 | ref | ref |
| Maternal age | <35 yrs | ref | ref |
|  | ≥35 yrs | 1.21[1.20,1.21] * | 1.27[1.26,1.28] * |
| Maternal education | below college | ref | ref |
|  | college or above | 1.07[1.07,1.07] * | 1.05[1.05,1.06] * |
| Marital status | single/divorce | 0.81[0.79,0.82] * | 0.71[0.69,0.73] * |
|  | married | ref | ref |
| Gestational age | every week increase | 0.89[0.89,0.89] * | 1.64[1.64,1.65] * |
| Parity | primipara | ref | ref |
|  | multipara | 1.29[1.28,1.29] * | 1.32[1.32,1.33] * |
| Prenatal visit | >8 times | ref | ref |
|  | ≤8 times | 0.91[0.91,0.91] * | 0.90[0.90,0.91] * |
| Scarred uterus | no | ref | ref |
|  | yes | 1.25[1.24,1.25] * | 1.26[1.25,1.26] * |
| Fetal sex | female | ref | ref |
|  | male | 0.95[0.95,0.95] * | 1.82[1.81,1.83] * |
| DIP | no | ref | ref |
|  | yes | 1.56[1.56,1.57] * | 1.89[1.87,1.90] * |
| Other complications | no | ref | ref |
|  | yes | 1.01[1.00,1.02] * | 1.16[1.16,1.17] * |

**: P<0.01*

**Supplementary Table 4. Descriptive statistics of maternal characters among the mediation sample**

| **Maternal characters** | | **Distribution^a^** | **Incidence n(%)^b^** | | |
| --- | --- | --- | --- | --- | --- |
|  |  |  | **DIP** | **LGA** | **Macrosomia** |
| All | | 10712534(100.00%) | 796409(7.43%) | 1851323(17.28%) | 777965(7.26%) |
| Region | east | 3127630(29.20%) | 290163(9.28%) | 575306(18.39%) | 236634(7.57%) |
|  | central | 4365719(40.75%) | 284713(6.52%) | 805476(18.45%) | 355669(8.15%) |
|  | west | 3219185(30.05%) | 221533(6.88%) | 470541(14.62%) | 185662(5.77%) |
| Area | north | 4368565(40.78%) | 282498(6.47%) | 910752(20.85%) | 419512(9.60%) |
|  | south | 6343969(59.22%) | 513911(8.10%) | 940571(14.83%) | 358453(5.65%) |
| Location | urban | 6429736(60.02%) | 644594(10.03%) | 1146792(17.84%) | 446368(6.94%) |
|  | rural | 4282798(39.98%) | 151815(3.54%) | 704531(16.45%) | 331597(7.74%) |
| Hospital level | level1 or level2 | 6075078(56.71%) | 303187(4.99%) | 1033369(17.01%) | 465834(7.67%) |
|  | level3 | 4637456(43.29%) | 493222(10.64%) | 817954(17.64%) | 312131(6.73%) |
| Maternal age | <35 yrs | 9346588(87.25%) | 610543(6.53%) | 1545362(16.53%) | 662889(7.09%) |
|  | above 35 yrs | 1365946(12.75%) | 185866(13.61%) | 305961(22.40%) | 115076(8.42%) |
| Maternal education | below college | 6229253(58.15%) | 336099(5.40%) | 1052046(16.89%) | 458999(7.37%) |
|  | college or above | 4272074(39.88%) | 436103(10.21%) | 763269(17.87%) | 305834(7.16%) |
|  | unknown | 211207(1.97%) | 24207(11.46%) | 36008(17.05%) | 13132(6.22%) |
| Prenatal visit | ≥8 times | 6023581(56.23%) | 580830(9.64%) | 1051043(17.45%) | 438416(7.28%) |
|  | <8 times | 4351727(40.62%) | 175937(4.04%) | 741893(17.05%) | 318410(7.32%) |
|  | unknown | 337226(3.15%) | 39642(11.76%) | 58387(17.31%) | 21139(6.27%) |
| Parity | primipara | 5725634(53.45%) | 406599(7.10%) | 858110(14.99%) | 386098(6.74%) |
|  | multipara | 4984036(46.53%) | 389657(7.82%) | 992724(19.92%) | 391688(7.86%) |
|  | unknown | 2864(0.03%) | 153(5.34%) | 489(17.07%) | 179(6.25%) |
| Marital status | single/divorce | 142823(1.33%) | 7412(5.19%) | 17929(12.55%) | 6529(4.57%) |
|  | married | 10567885(98.65%) | 788867(7.46%) | 1833056(17.35%) | 771309(7.30%) |
|  | unknown | 1826(0.02%) | 130(7.12%) | 338(18.51%) | 127(6.96%) |
| Scared uterus | no | 8877406(82.87%) | 615275(6.93%) | 1423127(16.03%) | 637956(7.19%) |
|  | yes | 1814228(16.94%) | 180553(9.95%) | 424514(23.40%) | 138701(7.65%) |
|  | unknown | 20900(0.20%) | 581(2.78%) | 3682(17.62%) | 1308(6.26%) |
| Gestational age | Median[Q1, Q3] | 39[38, 40] | 39[38, 39] | 39[38, 40] | 40[39, 40] |

Excluding SGA and women with one of the following complication: hypertensive disorder, placental disease (placenta previa and placenta accreta), intrahepatic cholestasis of pregnancy, thyroid disorder, heart disease, liver disease, kidney disease, lung disease, HIV, desmosis and cancer. N=10,712,534

a:distribution of each maternal character

b:incidence of DIP, LGA or macrosomia in each maternal character.

| A |  |
| --- | --- |
| B |  |

**Supplementary Figure 1. LGA and Macrosomia as the mediator in the association between DIP and obstetric hemorrhage from 2012 to 2021**

**Supplementary Table 5.** Crude results of LGA or macrosomia as the mediator through the association between DIP and obstetric hemorrhage

| **Effect decomposition** | **LGA** | | **Macrosomia** | |
| --- | --- | --- | --- | --- |
|  | **eRR** | **PM** | **eRR** | **PM** |
| TE | 0.49[0.47,0.51] | 5.43%[4.78%,6.07%]* | 0.49[0.47,0.51] | 3.06%[2.65%,3.47%]* |
| PDE | 0.46[0.45,0.48] |  | 0.48[0.46,0.49] |  |
| TIE | 0.03[0.02,0.03] |  | 0.02[0.01,0.02] |  |

*：*P<0.01*

TE: total effect

PDE: pure direct effect

TIE: total indirect effect

eRR: excess relative risk

PM: proportion mediated

**Supplementary Table 6.** LGA as the mediator through the association between DIP and obstetric hemorrhage using different standard

| **Effect decomposition** | **National fetal growth standard** | | **INTERGROWTH-21st standard** | |
| --- | --- | --- | --- | --- |
|  | **eRR** | **PM** | **eRR** | **PM** |
| TE | 0.21[0.19,0.22] | 12.10%[10.76%,13.45%]* | 0.21[0.19,0.22] | 11.89%[10.57%,13.22%]* |
| PDE | 0.18[0.17,0.20] |  | 0.18[0.17,0.20] |  |
| TIE | 0.02[0.02,0.03] |  | 0.02[0.02,0.03] |  |

*：*P<0.01*

TE: total effect

PDE: pure direct effect

TIE: total indirect effect

eRR: excess relative risk

PM: proportion mediated
